# Supplementary material for: How to lead health care workers during unprecedented crises: A qualitative study of the COVID-19 pandemic in Connecticut, USA
Source: PLoS One. 2021 Sep 14;16(9):e0257423. doi: 10.1371/journal.pone.0257423 (PMC8439455; doi:10.1371/journal.pone.0257423)
Supplement: S1 File — (DOCX) [file pone.0257423.s001.docx]

Dear (Name),

Thank you so much for your interest in participating in this study! Participation involves a 45-minute virtual interview via Zoom about your experiences as a health care worker during the COVID-19 pandemic.

If you are interested in proceeding, could you please answer the following questions?

1. What type of health care worker are you (e.g. physician, nurse, patient care assistant, respiratory therapist)?
2. Did you have in-person encounters with patients as a health care worker anytime from March 2020 until present (yes/no)?
3. Where do you work (e.g. hospital, clinic, both hospital and clinic, nursing home, retirement home)?
4. (If appropriate) What were the settings in which you practiced (e.g. ICU, floor, subspecialty)?
5. Which state/country do you work as a health care worker?
6. Approximately how many COVID-positive patients did you care for over the past few months?

Could you please provide four 1-hour blocks of time between 9 AM-7 PM over the next two weeks that you are available to be interviewed if you are eligible for the study?

Thank you!

Sincerely,
