# Supplementary material for: How to lead health care workers during unprecedented crises: A qualitative study of the COVID-19 pandemic in Connecticut, USA
Source: PLoS One. 2021 Sep 14;16(9):e0257423. doi: 10.1371/journal.pone.0257423 (PMC8439455; doi:10.1371/journal.pone.0257423)
Supplement: S2 File — (DOCX) [file pone.0257423.s002.docx]

|  | **Questions** |
| --- | --- |
|  | 1. Tell me about your role in the hospital |
|  | 1. Tell me about what it has been like to work during the COVID-19 pandemic. |
|  | 1. How, if at all, did your scope of work change during the COVID-19 pandemic? |
|  | 1. Can you tell me a little bit about how your hospital/department has responded to COVID-related risks? |
|  | 1. Is there anything else your institution could have done to support you? |
|  | 1. What is the situation with PPE at your job? How did you navigate this situation? |
|  | 1. Were there times you had symptoms similar to COVID? What was that like for you? Were there times you considered missing work to self-isolate? What was that like for you? Can you describe the response of your employer/co-workers? |
|  | 1. What have your relationships with your colleagues been like during the pandemic? |
|  | 1. How, if at all, did the pandemic impact your relationship with your patients? |
|  | 1. How, if at all, did/does your work impact your relationship with your family, friends, community? |
|  | 1. How would you describe other people’s attitudes towards you as a healthcare worker? |
|  | 1. What has been the most challenging thing for you as a healthcare worker over the last few months of this pandemic? |
|  | 1. This was a challenging time, what helped you get through it? |
|  | 1. What is your perspective on recent “reopening”? |
|  | 1. How will this impact your training/future? |
|  | 1. Did you encounter any resource limitations? If so, what? |
|  | 1. Anything else you would like to add? |
|  |  |
|  | **Demographics** |
|  | 1) How old are you? |
|  | 2) What is your specialty (if any)? Are you a resident (yes/no)? |
|  | 3) How many years have you been practicing as a ____ (role e.g. resident, doctor, nurse, etc.)? For attendings including residency and just as an attending |
|  | 4) Are you married or single? |
|  | 5) Do you have any children? If so, how many? |
|  | 6) What state did you practice medicine during the pandemic? |
|  | 7) Did you work as an outpatient, inpatient or both during the pandemic? |
|  | 8) How many known COVID-19 patients did you provide care to during the pandemic? [ ] 0 [ ]1-5 [ ]6-10 [ ]11-20 [ ]> 20 |
|  | 9) What race or ethnicity do you most identify with? |
